# Targeting IL-4 and IL-13 Receptors on Eosinophils in CRSwNP Patients: The Clinical Efficacy of Dupilumab

**DOI:** 10.3390/jpm13091404

**Published:** 2023-09-19

**Authors:** Giovanna Lucia Piazzetta, Nadia Lobello, Emanuela Chiarella, Alberta Rizzuti, Corrado Pelaia, Girolamo Pelaia, Nicola Lombardo

**Affiliations:** 1 Otolaryngology Head and Neck Surgery, Department Medical and Surgical Sciences, University “Magna Græcia”, 88100 Catanzaro, Italy; giovannapiazzetta@hotmail.it (G.L.P.); nadialobello@gmail.com (N.L.); alberta.rizzuti@guest.policlinicogemelli.it (A.R.); 2 Laboratory of Molecular Haematopoiesis and Stem Cell Biology, Department of Experimental and Clinical Medicine, University “Magna Græcia”, 88100 Catanzaro, Italy; emanuelachiarella@unicz.it; 3 Department of Health Sciences, University “Magna Græcia” of Catanzaro, 88100 Catanzaro, Italy; pelaia.corrado@gmail.com (C.P.); pelaia@unicz.it (G.P.)

**Keywords:** chronic rhinosinusitis, nasal polyps, inflammatory disease, type 2 inflammation, dupilumab, cytokines, numeric rating scale (NRS), Sino Nasal Outcome Test-22 (SNOT-22), nasal polyps score (NPS), peripheral eosinophilia, IgE

## Abstract

Chronic rhinosinusitis with nasal polyposis (CRSwNP) is an inflammatory disease linked to type 2 inflammation. Several biologics have demonstrated therapeutic potential for the treatment of this pathology in which IL-4, IL-5 and IL-13 represent the major cytokines involved in the control of eosinophilic respiratory inflammation. 25% of CRSwNP patients relapse after the use of oral glucocorticoids or after surgery and often require several surgeries during their lifetime. In our study we enrolled 14 patients, 11 male and 3 female. The inclusion criteria were: age ≥ 18 years; confirmed diagnosis of chronic rhinosinusitis with severe nasal polyposis; disease severity with NPS Nasal Polyposis Endoscopic Score total score ≥ 5 and/or SNOT-22 ≥ 50; previous treatment failure due to lack of efficacy or discontinuation of systemic corticosteroid therapy and/or non-response or recurrence following surgery. The results presented in this study showed the ability of Dupilumab to improve all the parameters analysed. In particular, statistically significant data were obtained for NPS, SNOT-22, NRS, and IgE in patients exposed to Dupilumab treatment for 24 weeks, highlighting the ability of Dupilumab to produce clinical benefit in CRWwNP patients. In light of these data, the administration of dupilumab every two weeks represents a valid clinical strategy that ENT specialists can adopt for the treatment of adults with inadequately controlled CRSwNP.

## 1. Introduction

Chronic rhinosinusitis (CRS) is an inflammatory disease of the mucous membrane of the nose and paranasal sinuses [1]. It is characterized by symptoms persisting for more than 12 weeks and, having a great impact on the life quality in the patients affected. CRS is responsible for more than one million surgeries a year worldwide [2]. There are two major types of CRS: the chronic rhinosinusitis with nasal polyps (CRSwNP), which in about 80% of cases and is supported by type 2 inflammation and, the chronic rhinosinusitis without nasal polyposis (CRSsNP) with a more heterogeneous inflammatory mechanism mainly associated with type 1 and 3 inflammation [3,4]. This pathology affects about 12% of the population in the USA, about 11% in Europe, 9–10% in Asia and 8.5% in Australia [5]. It most frequently affects adults and in particular people over 40 years old. Men are more affected than women who appear to have more severe symptoms and see greater impairment on quality of life [6]. Approximately 50% of patients with nasal polyposis are also affected by bronchial asthma, 72% by allergic rhinitis, 10–20% by NSAID-ERD (Nonsteroidal anti-inflammatory drug-exacerbated respiratory disease), and approximately 8% suffer from atopic dermatitis [7]. The diagnosis of CRS with or without nasal polyposis is based on symptoms and objective evidence of pathology. According to the diagnostic criteria of EPOS 2020 (European Position Paper on Rhinosinusitis and nasal polyps 2020), for the diagnosis of CRSwNP, two or more of the following symptoms must be found, (of which at least one between nasal congestion/obstruction or anterior rhinorrhea/rear must be compulsorily present): nasal obstruction or congestion; anterior or posterior rhinorrhea (Post Nasal Drip); with or without facial pain or pressure; with or without hypo/anosmia [6]. The symptoms must be accompanied by the objective finding of endoscopic signs of nasal polyposis such as mucous discharge from the middle meatus or edema or obstruction of the middle meatus and/or signs of inflammatory alteration of the mucosa at the osteomeatal level highlighted on Computed Tomography. Nasal polyposis symptoms have a major impact on both social life and mental health. One of the most important consequences of the disease are sleep disturbances which will lead the patient to wake up already tired or to tire easily during the day [8]. The evaluation of the patient foresees a first diagnostic phase, with estimation of the sino-nasal symptomatology, objectifying the symptom with numerical values attributed by the patient himself; a subsequent phase in which social/occupational functioning, emotional consequences and overall satisfaction with care are assessed. This second phase aims to estimate the impact of the disease on the Quality of Life QoL [9]. CRSwNP produces less pain and more facial pressure; it is more likely to cause anosmia, less likely to respond to antibiotics, and more likely to respond to corticosteroids [1]. Nasal polyps, whose etiology is not fully understood, are benign and painless excrescences, with a translucent and gelatinous appearance that originate from the ethmoid sinuses and affect the nasal mucosa and paranasal sinuses. Among the many factors involved in the development and progression of polyps, anatomical disorders, genetic factors, infections caused by viruses, bacteria, fungi, as well as allergic rhinitis and non-allergic inhalants assume a relevant importance [10]. The formation of the nasal polyp involves histologically well-identified mechanisms which are the rupture of the mucosal epithelium, the proliferation of fibrous tissue through the damaged epithelium, the accumulation of extracellular matrix (ECM) with edema and the proliferation of a granular tissue formed by thin-walled vessels and infiltration of inflammatory cells [11]. However, the mere determination of the phenotype, with or without polyps, does not allow us to delve into the complexity of the underlying pathophysiological mechanisms and to frame chronic rhinosinusitis from the point of view of a medicine of precision [12]. It is necessary to identify endotypes, i.e., biological subtypes characterized by similar cell types and/or similar inflammatory and molecular patterns, which can be targeted by a personalized therapy, the so-called “tailored therapy” [13,14]. Endotyping has led to the use of innovative biological drugs that are revolutionizing the treatment of patients suffering from chronic rhinosinusitis [15]. Traditionally, the medical treatment of chronic rhinosinusitis with nasal polyposis uses intranasal and/or systemic corticosteroids and antibiotics. Although divergences in responses to therapy have been observed especially with regard to the duration and maximum dose of systematic steroids that place patients at high risk of adverse events [16]. Surgery is effective in unblocking the nasal passages, restoring respiratory function as well as possible and allowing corticosteroids to reach the sinus mucosa. Since the goal of surgery is to improve the severity of the patient’s symptoms, the decision to operate should be made only in patients with symptomatic disease, except for patients with current or impending complications. Biologics represent the last frontier for CRSwNP treatment [17]. Most patients with chronic rhinosinusitis with nasal polyps have a type 2 inflammatory pattern characterized by eosinophilia and elevated levels of interleukin-4, interleukin-5, and interleukin-13 [18]. These cytokines have been extensively studied regarding their role in eosinophilic respiratory inflammation in CRSwNP [19]. Several biologics were approved for the management of severe asthma, and resulted optimal therapeutics potential for the treatment of CRS, with particular regard to patients with CRS and nasal polyposis (CRSwNP). The role of biologics in CRSwNP treatment requires careful consideration of different factors, including disease severity, risk of polyp recurrence with medical or surgical treatment, patient preferences and goals, safety, and costs–effectiveness ratio [20]. Currently, phase 3 studies, focused on biologics targeting IL-5 and eosinophil pathway, IgE and mast cell activation or IL-4/IL-13 pathway [21]. Among them the fully humanized IgG4 monoclonal antibody Dupilumab (DUPIXENT, Sanofi and Regeneron) targets the α subunit of the IL-4 receptor (IL-4Rα) which is shared by IL-4 type I and IL-4/IL-13 type II receptor complexes [22]. The efficacy and safety of Dupilumab in CRSwNP patients previously treated with systemic corticosteroids, surgery, or both, was evaluated by SINUS-24 and SINUS-52 studies, two multinational, multicenter, randomized and double-blind studies [23]. Dupilumab is now approved as an add-on therapy for severe asthma, it is also licensed for the treatment of nasal polyposis and atopic dermatitis [24]. In chronic rhinosinusitis with nasal polyposis, subcutaneously administered dupilumab is the first biologic therapy to be approved for the treatment of adults with inadequately controlled CRSwNP in the EU and USA [25]. Th2 and Group 2 Innate Limphoid cells (ILC2) release large amounts of IL4, IL13 and IL5. The first small molecule induces immunoglobulin class switching, thereby stimulating B lymphocytes to synthesize IgE. IL-13 cooperates with IL-4 in inducing IgE production and is also responsible for bronchial hyperreactivity, goblet cells metaplasia, proliferation of fibroblasts and airway smooth muscle cells. In addition it was demonstrated a role for Dupilumab in regulating the expression of the iso-inducible form of nitric oxide synthase (iNOS), thereby increasing NO emission from airway epithelial cells, as well as the levels of exhaled nitric oxide fraction (FeNO) [26]. IL-4 and IL-13 share common signaling pathways, activated by the binding of both cytokines to a receptor complexes especially the α subunit of the IL-4 receptor (IL-4Rα). Therefore, the subsequent dimerization of the receptor is responsible for the pathophysiological effects of IL-4 and IL-13. In this molecular context, Dupixent plays a leading role and exerts its activity by blocking the shared receptor for interleukin-4 and interleukin-13, which are two factors responsible for type 2 inflammation [27].

## 2. Materials and Methods

### 2.1. Patients Enrollment and Dupilumab Treatment

In this retrospective study were enrolled a total of 15 patients whose 14 subjects have completed the treatment for at least 6 months, while one was exposed to only two Dupilumab administrations for reasons not attributable to adverse events to the therapy but rather to compliance issues on a psycho-emotional basis. This study met the standards of Good Clinical Practice and the principles of the Declaration of Helsinki. All patients signed a written informed consent. The study was also carried out in agreement with a statement from the local Ethical Committee of Calabria Region (Catanzaro, Italy; document 182—20 May 2021).

The definition of the clinical outcomes was made on the cohort of 14 patients consisting of 11 males and 3 females, aged between 31 and 79 years, affected by chronic rhinosinusitis with nasal polyposis. Within this group of patients, 8/14 had a clinical diagnosis of asthma, whose 2/8 suffered from moderate asthma and 6/8 from mild asthma. In this group 6 patients suffered from inhalant allergy and 3 had NSAID-ERD. Patients were treated with Dupixent^®^ (Dupilumab), 300 mg solution for injection in pre-filled pen, by subcutaneous injection every 2 weeks. Each single-use pre-filled pen contains 300 mg of Dupilumab in 2 mL of solution (150 mg/mL). The treatment was associated with topical intranasal corticosteroid therapy with mometasone furoate nasal spray (2 administrations per day for a total of 200 mg/day). The inclusion criteria in the study were: (1) age ≥ 18; (2) confirmed diagnosis of chronic rhinosinusitis with severe nasal polyposis; (3) disease severity with Nasal Polyposis Endoscopic Score (NPS) total score ≥ 5 and/or (4) SNOT-22 ≥ 50; (5) previous treatment failure due to lack of efficacy or discontinuation of systemic corticosteroid therapy and/or non-response or recurrence following surgery. Within the clinical group examined, 13 patients underwent at least one FESS surgery, while only one patient was not operated on due to a high anesthetic risk which made it impossible to carry out the surgical therapy and was directed directly to biological therapy. Patients evaluation was performed using the following parameters: endoscopic nasal polyps score (NPS), peripheral eosinophilia, total IgE, impact QoL through the Sino Nasal Outcome Test (SNOT-22)—Washington University—St. Louis, Missouri and finally the general impact of the disease using a numerical rating scale (NRS = numeric rating scale).

### 2.2. SNOT-22

The Sino Nasal Outcome Test (SNOT-22)—Washington University—St. Louis, Missouri allows to estimate the Quality of Life (QoL) in CRwNP patients according 22 items. A score ranging from 0 (symptom not perceived) to 5 (worst possible perception of the symptom) is assigned for each item. The total score ranges from 0 to 110. A high score is associated with a worse outcome.

### 2.3. NPS

To evaluate the nasal polyps score (NPS) according to the polyp grading system, a nasal endoscopy was performed using an OLYMPUS ENF TYPE-GP fiberscope. The NPS score is based on the size of the polyp, which is scored from 0 to 4 for each nasal cavity. The score obtained is directly proportional to the severity of the pathology.

### 2.4. NRS

The numeric rating scale (NRS) was used to evaluate how the patients subjective perceive the disease. A score ranging from 0 (“no problem”) to 10 (“worst possible perception”) is assigned. The values are divided into three ranges: (1) 0–3 = mild symptoms; (2) 4–7 = moderate symptoms; (3) 8–10 = severe symptoms [28].

### 2.5. Statistical Analysis

Statistical analysis was performed using Microsoft Excel 2019, software. All data are expressed as means ± SD. Student’s *t*-tests was used to compare variables when considered appropriate. For all analyses were considered statistically significant the following *p* values: * *p* < 0.05; ** *p* < 0.001; *** *p* < 0.0001.

## 3. Results

### 3.1. Distribution of CRSwNP Patients by Gender and Age

**Figure 1:**
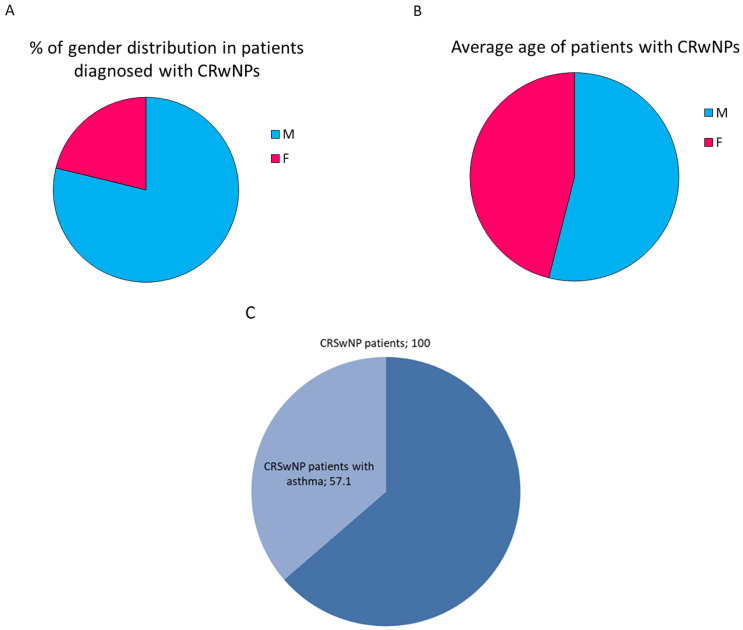
Pie charts represent (**A**) the percentage of gender distribution and (**B**) the avarage age in patients with CRwNPs and (**C**) the percentage of CRSwNP patients with asthma.

To evaluate the clinical efficacy of Dupilumab 14 patients diagnosed with CRwNPs have been enrolled. Specifically, 78% were males and the 21% were famales, Figure 1A. The mean age of the enrolled patients was 62.54 for men and 53.3 for woman, Figure 1B. The median value of the entiere analysed group of patients was 61.5. Analithically, the demographic data of CRSwNP patients enrolled in this study are reported in Table 1. In addition the 57.1% of enrolled CRSwNP patients were asthmatic (Figure 1C).

### 3.2. Determination of SNOT-22, NPS and NRS in Patients Diagnosed CRSwNP and Treated with Dupilumab for 24 Weeks

**Figure 2:**
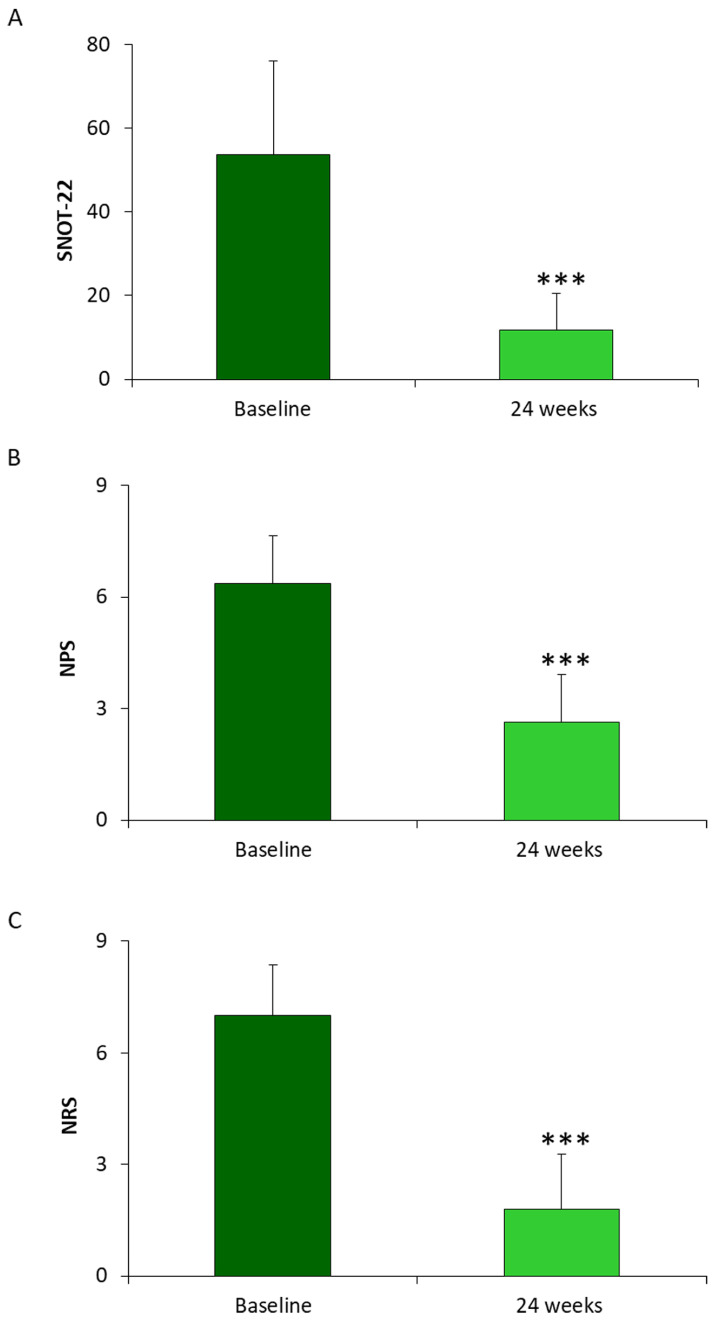
Histograms represent the mean values of (**A**) SNOT-22, (**B**) NPS and (**C**) NPR evaluated in 14 patients with CRwNPs treated with Dupilumab 300 mg for 24 weeks. The data were compared to the mean values at the baseline levels. Data are represented as means ± SD from 14 patients (*** *p* < 0.0001).

Patients exposed to Dupilumab treatment for 24 weeks were evaluated for SNOT-22, NPS and NRS parameters. SNOT-22 was reduced 5-fold in patients who completed treatment with Dupilumab compared to initial diagnosis. The mean SNOT-22 value at time 0 was 53.64 ± 22.39 while at 24 weeks it was 11.86 ± 8.73. The severity of CRSwNP measured by NPS decreased of about 2.4 fold after 24 weeks compared to initial pathologic condition. In particular, the mean value of the Nasal Polyps score at baseline was 6.36 ± 1.28 while after 24 weeks a value of 2.63 ± 1.33 was obtained. The analysis of NRS parameter indicates an improvement of individuals’ perception of the disease, indeed dupilumab treatment positively impacted on NRS by reducing values of 3.9 fold in the patients considered in this study. The NRS scale at time at baseline showed a mean value of 7 ± 1.36, resulting instead equal to 1.79 ± 1.48 at 24 weeks, Figure 2.

### 3.3. Determination of Peripheral Eosinophilia and Total IgE in Patients Diagnosed CRSwNP Treated with Dupilumab for 24 Weeks

**Figure 3:**
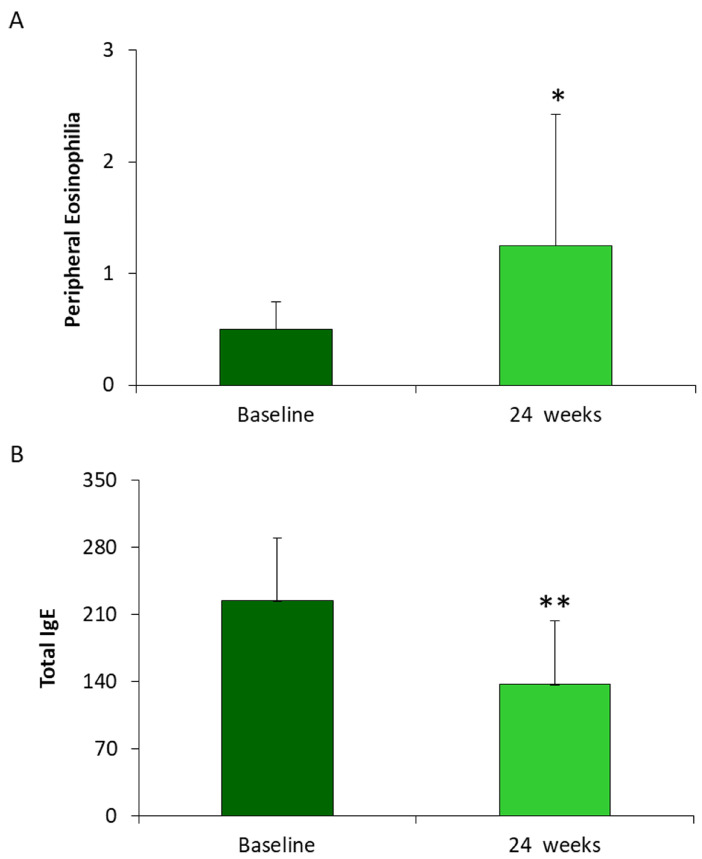
Histograms represent the mean values of (**A**) Peripheral Eosinophilia and (**B**) total IgE evaluated in 14 patients with CRwNPs treated with Dupilumab 300 mg for 24 weeks. The data were compared to the mean values at the baseline levels. Data are represented as means ± SD from 14 patients (* *p* < 0.05; ** *p* < 0.001).

Peripheral eosinophilia and total IgE were analyzed after 24 weeks treatment. With regard to the eosinophilia parameter, a significant increase of about 2.5 times in patients undergoing treatment is appreciable, compared to baseline. The mean value of peripheral eosinophils was of 0.49 ± 0.25 cells/μL at baseline and 1.25 ± 1.18 cells/μL at 24 weeks. On the other hand, the data relating to total blood IgE decreased from a value of 224 ± 65 kU/L at baseline, to a value of 136± 66.5 kU/L at 24 weeks, Figure 3.

### 3.4. Effectiveness of Dupilumab Administration in 14 CRSwNP Patients

**Figure 4:**
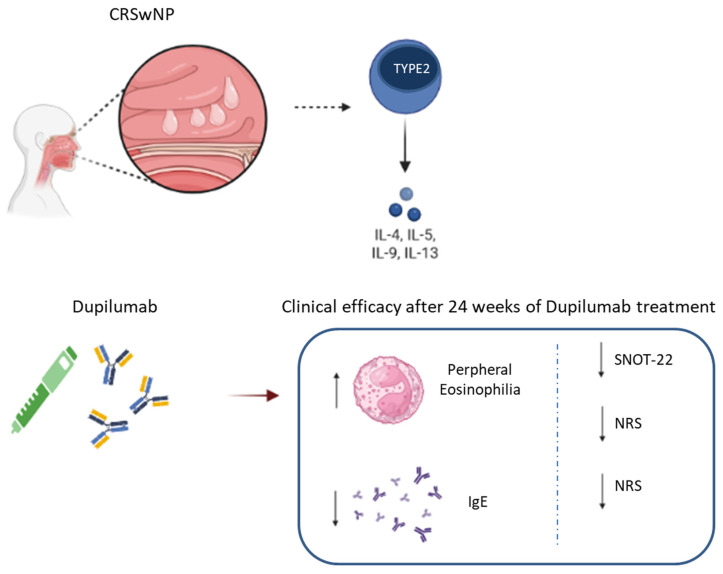
Schematic representation of the effects of dupilumab on 14 patients with CRSwNP. CRSwNP is an inflammatory disease in which Th2 cells play a central role by releasing cytokines, such as IL-4, IL-5, IL-9, IL-13. Dupilumab, a humanized monoclonal antibody, targeting the IL-4 receptor affects the inflammatory pathophysiology of the respiratory tract improving the quality life of patients. An increase in peripheral eosinophilia and a decrease in total IgE were observed when patients were exposed to 24 weeks of dupilumab dosing. Treatment with Dupilumab for 24 weeks evoked a marked reduction in the Nasal rating score (NRS), Sino Nasal Outcome Test-22 (SNOT-22), Nasal Polyps Score (NPS). The diagram was made by Biorender.

Our study demostrated Dupilumab 300 mg administered subcutaneously every two weeks is a valid add-on treatment option for adults with inadequately controlled CRSwNP. After 24 weeks of Dupilumab treatment, the analyzed patients experienced remarkable improvement in SNOT-22, NPS and NRS parameters; while resulted in a significant reduction of peripheral eosinophilia and in the enhancement of total IgE (Figure 4).

## 4. Discussion

Dupilumab is a human IgG4 monoclonal antibody that recognizes the α subunit of the interleukin IL-4 receptor (IL4Rα). Since IL-4Rα activation is utilized by both IL-4 and IL-13 for the transduction of molecular events responsible for pathophysiological effects, Dupilumab acts as a dual antagonist of these two cytokines, blocking the signaling transduction IL-4 and IL-13 dependent. This mechanism of action determines an arrest of the reparative remodeling events typical of chronic rhinosinusitis with nasal polyps based on type 2 inflammation [22]. During 2021 EMA and AIFA authorized the use of Dupilumab in type 2 nasal polyposis, therefore, since January 2021, for the first time in Italy, Dupilumab has become part of the therapeutic treatment of nasal polyposis [29]. SINUS-24 and SINUS-52 studies indeed demonstrated that the administration of Dupilumab (300 mg every 2 weeks) to standard therapy in adults with severe CRSwNP was evaluated in parallel groups and compared to patients riceiving placebo. SINUS-24 was conducted in 67 centers in 13 countries while SINUS-52 in 117 centers in 14 countries. Eligible patients were 18 years of age or older with bilateral CRSwNP and persistent symptoms despite intranasal corticosteroid use; in addition, some subjects had received systemic corticosteroids in the previous 2 years or had undergone nasosinusal surgery. Randomisation was stratified by asthma or NSAID-exacerbated respiratory disease status at the time of screening, prior surgeries, and country of origin. Patients with or without comorbid asthma were included. The data from the studies support the benefits of adding dupilumab to the standard therapy in patients with CRSwNP as an innovative approach in the treatment of the full spectrum of clinical manifestations of the disease, the underlying predominantly type 2 inflammatory condition, as well as comorbidities of the lower airways often associated with type 2 and Non Allergic Rhinitis (NAR) [30]. Dupilumab treatment reduced nasal polyp size, improved sinus opacification, and health-related quality of life (HR-QOL). It also relieved core symptoms of CRSwNP such as nasal congestion or obstruction, nasal discharge and loss of smell, and reduced the use of systemic corticosteroids and the need for surgery, providing effective treatment for patients with severe CRSwNP who would otherwise be left with few treatment options. The drug was generally well tolerated and demonstrated an acceptable level of safety [29]. Conjunctivitis was reported in seven patients who received dupilumab and one patient who received placebo; none of these patients had serious, severe, or associated symtoms with treatment discontinuation. Four patients experienced eosinophilia with clinical symptoms reported as treatment-emergent adverse events; specifically, one patient experienced eosinophilic granulomatosis with polyangiitis (EGPA) while receiving dupilumab; one patient experienced eosinophilia associated with arthralgia, asthma exacerbation, and insomnia while receiving dupilumab; one patient experienced EGPA more than 300 days after a single dose of dupilumab [31,32]. The data reported in this paper therefore represent a first evaluation of the clinical effects of Dupilumab in a group of patients affected by severe CRSwNP. The concept of disease severity is defined on the basis of a series of parameters that correspond to those used in the definition of the treatment plan [33]. In the patients analyzed in our study, the severity of the disease is evidenced by the high value of the nasal polyp score at time T0, the clinical history usually associated to comorbidities such as bronchial asthma, the recurrence of the disease after endoscopic nasal surgery, and again by the high value of total blood IgE and the basic blood eosinophilia level. Patients admitted to Dupilumab therapy had to satisfy specific requirements, first of all the lack of disease control after surgery or despite receiving a double course of oral corticosteroids in the last year. Here the results showed that the use of this monoclonal antibody improved all the parameters considered in our investigation. Statistically significant data were obtained for the nasal polyp score (NPS) which, more markedly than reported in the SYNUS 24 and SYNUS 52 clinical trials, went from an average value of 6.36 ± 1.28 at time T0 to one of 2.63 ± 1.33 at time T1. SNOT-22 also considerably improved, increased from a mean value at time T0 of 53.64 ± 22.39 to a final value at T1 of 11.86 ± 8.73. The qualitative improvement in the life of our patients is further confirmed by the reduction of the value of the Numeric Rating Scale on the perception of the disease which has decreased from the T0 value of 7 ± 1.36 to the final value at time T1 of 1.79 ± 1.48.

The results here presented are supported by a variety of real-life studies. Peters et al. demonstrated the efficacy of Dupilumab in CRSwNP patients with and without allergic rhinitis. Specifically, the administration of the monoclonal antibody improved all considered parameters (SNOT-22, NPS and LMK) and reduced the levels of type 2 biomarkers compared to placebo [34]. In addition, our data are consistent with the longitudinal study by Florian Jansen et al. The authors studied 40 patients treated with Dupilumab for 13 months highlithing a linear dependence of some parameters, including NPS and SNOT-22, under therapy. Also Sniffin’ Sticks-12 identification test (SSIT) used for olfactometry evaluation resulted improved [35]. These effects were also proved by The randomized controlled trial from Joaquim Mullol. They focused on a cohort of 724 patients (286 placebo, 438 dupilumab) demonstrating that the treatment with dupilumab was effective in relieving symptoms of severe CRSwNP and most interestingly produced a more pronounced significant improvement in sense of smell at 24 weeks compared to baseline [36]. In addition, the efficacy of dupilumab was evaluated in a cohort of patients with severe CRSwNP producing improvement in nasal obstruction and olfaction both in naïve patients and in those sujected to endoscopic sinus surgery in the past [37]. Intriguing, a recent multicentric real-life study performed on 648 patients with severe uncontrolled CRSwNP, showed effectiveness of dupilumab 300 mg self-administered subcutaneously every 2 weeks as add-on therapy to intranasal corticosteroids. In these patients, Dupilumab treatment resulted in polyp size reduction, improvement of quality of life, severity of symptoms, nasal congestion, and smell function [38]. With regard to eosinophilia, our data shown a significant increase in the mean values, which pass from 0.49 ± 0.25 cells/μL to 1.25 ± 1.18 cells/μL. This increase in eosinophils, already known during clinical trials, is the expression of the mode of action of the drug which, by inhibiting IL-13, reduces tissue recruitment of eosinophil cells, causing eosinophil cell trafficking inhibition. Dupilumab-induced hypereosinophilia is a clinical complication that the clinic must manage. To date, algorithms have been proposed to study Dupilumab-induced hypereosinophilia and to adequately manage potential co-morbidities related to the increase in eosinophil numbers in order to avoid drug discontinuation [39]. As regards the mean value of IgE, there was a statistically significant reduction of the same, which went from an initial value of 224 ± 65 kU/L to a final value of 136 ± 66.5 kU/L. This widely expected result is the consequence of the switching inhibition in IgE production related to IL-4 as well as its action in converting B lymphocytes into plasma cells and subsequently IgE production. Our study highlights the ability of Dupilumab in obtaining a clinical benefit within 24 weeks of treatment. This important aspect has already been reported in a real life study by Pelaia G. et al. in which the authors demonstrated the valuable effect of Dupilumab in both allergic and non allergic asthmatic patients affected also by nasal polyposis [23]. The SNOT-22 and the NRS show a significant improvement in the olfactory function with great patient satisfaction, which is difficult to find in other medical therapies of the otolaryngology specialty. These evidences provide new criteria to re-evaluate the indications for the administration of Dupilumab, which cannot be traced back to a simple evaluation of the size of the polyp but more ethically to an improvement of the emotional and qualitative parameters of the patient’s life. The absence of side effects resulting from the Dupilumab treatment in the analyzed group of patients is significant, despite in the literature are reported cases of hypersensitivity, keratoconjunctivitis, nasopharyngitis and eczema herpes [40]. These complications were not found in our patients, confirming the excellent safety profile of Dupilumab. A significant improvement in the outcomes of the disease and above all an extraordinary improvement in the patient’s quality of life has been achieved. In our study, the usefulness of the correctly indicated drug was further validated in real-life conditions. The new generation otolaryngologist will be called upon to manage and select the indications for the use not only of Dupilumab but also of the other biological drugs that will enter the therapeutic handbook. The identification of new therapeutic targets and the development of new biological therapies suggest a real revolution in CRSwNP treatment with particular attention to the resolution of the symptoms felt by the patient. These typical disease signatures until now have represented an unmet need for common therapies. Surely a non-secondary fact is the economic sustainability of pharmaceutical expenditure for these pathologies. The medical specialist is called to make appropriate and ethically aware choices in the light of the priorities of therapeutic indications and the availability of economic resources. With the advent of biologics, scientific research has moved towards the discovery of CRwNP biomarkers able to support the path of personalization of care.

## 5. Conclusions

Our study demostrated Dupilumab 300 mg administered subcutaneously every two weeks is a valid add-on treatment option for adults with inadequately controlled CRSwNP. After 24 weeks of Dupilumab treatment, the analyzed patients experienced remarkable improvement in SNOT-22, NPS and NRS parameters; while resulted in a significant reduction of peripheral eosinophilia and in the enhancement of total IgE (Figure 4). It has proven to be effective, safe and with a quick onset of action. No adverse effects were reported in the group of analyzed patients, allowing us to appreciate a good safety profile. A significant improvement in the outcomes of the disease and above all an extraordinary improvement in the patient’s quality of life has been achieved. In our study, the usefulness of the correctly indicated drug was further validated in real-life conditions. The new generation otolaryngologist will be called upon to manage and select the indications for the use not only of Dupilumab but also of the other biological drugs that will enter the therapeutic handbook. The identification of new therapeutic targets and the development of new biological therapies suggest a real revolution in CRSwNP treatment with particular attention to the resolution of the symptoms felt by the patient. These typical disease signatures until now have represented an unmet need for common therapies. Surely a non-secondary fact is the economic sustainability of pharmaceutical expenditure for these pathologies. The medical specialist is called to make appropriate and ethically aware choices in the light of the priorities of therapeutic indications and the availability of economic resources. With the advent of biologics, scientific research has moved towards the discovery of CRwNP biomarkers able to support the path of personalization of care. After 24 weeks of Dupilumab treatment, the analyzed patients experienced remarkable improvement in SNOT-22, NPS and NRS parameters; while resulted in a significant increase of peripheral eosinophilia and in the reduction of total IgE. It has proven to be effective and safe and with a quick onset of action. In our study, the usefulness of the correctly indicated drug was further validated in real-life conditions.

## 6. Originality and Study Limitation

Considering that nasal polyposis is a severe and recurrent disease with considerable discomfort to patients, we have shown that treatment with Dupixent for 24 weeks strengthens the NPS data obtained from the Sinus trials and therefore produces an improving effect on the patient’s quality of life. However, the limitation of this study could be represented by a small number of enrolled patients.

## Figures and Tables

**Table 1:** Demographic data of CRSwNP patients enrolled in this study to evaluate the clinical efficacy of Dupilumab.

Patients	Sex	Age
1	M	52
2	M	75
3	F	61
4	F	31
5	M	47
6	F	68
7	M	79
8	M	73
9	M	59
10	M	54
11	M	66
12	M	59
13	M	62
14	M	62

## Data Availability

Not applicable.
